# The Flexibility of Conceptual Pacts: Referring Expressions Dynamically Shift to Accommodate New Conceptualizations

**DOI:** 10.3389/fpsyg.2016.00561

**Published:** 2016-04-25

**Authors:** Alyssa Ibarra, Michael K. Tanenhaus

**Affiliations:** ^1^Department of Brain and Cognitive Sciences, University of RochesterRochester, NY, USA; ^2^Department of Linguistics, School of Arts & Sciences, University of RochesterRochester, NY, USA

**Keywords:** referential expressions, conceptual pacts, entrenchment, targeted language game, interactive conversation

## Abstract

In a classic paper, Brennan and Clark argued that when interlocutors agree on a name for an object, they are forming a temporary agreement on how to conceptualize that object; that is, they are forming a conceptual pact. The literature on conceptual pacts has largely focused on the costs and benefits of breaking and maintaining lexical precedents, and the degree to which they might be partner-specific. The research presented here focuses on a question about conceptual pacts that has been largely neglected in the literature: To what extent are conceptual pacts specific to the local context of the interaction? If conceptual pacts are indeed temporary, then when the local context changes in ways that are accessible to participants, we would expect participants to seamlessly shift to referential expressions that reflect novel conceptualizations. Two experiments examined how referential forms change across context in collaborative, task-oriented dialog between naïve participants. In Experiment 1, names for parts of an unknown object were established in an “item” identification stage (e.g., a shape that looked like a wrench was called “the wrench”). In a second “build” stage, that name was often supplanted by an object-oriented name, e.g., the “leg.” These changes happened abruptly and without negotiation. In Experiment 2, interlocutors manipulated clip art and more abstract tangram pictures in a “slider” puzzle to arrange the objects into a target configuration. On some trials moving an object revealed a picture that could be construed as a contrast competitor, e.g., a clip art picture of a camel after “the camel” had been negotiated as a name for a tangram shape, or vice versa. As would be expected, modification rates increased when a potential contrast was revealed. More strikingly, the degree to which a name had been negotiated or the frequency with which it had been used did not affect the likelihood that the revealed shape would be considered as a potential contrast. We find little evidence that names that are introduced as part of a conceptual pact persist when either the task goals or informational needs change. Rather, conceptual pacts are fluid temporary agreements.

## Introduction

There is a many-to-many mapping between names and potential referents. A picture of a Bernese Mountain Dog could be referred to as “the dog”, “the Bernese Mountain Dog,” “the Berner,” etc. Furthermore, names can be assigned on the basis of the properties of the object (e.g., “the brown dog”), assigned with respect to a particular referential domain (e.g., “the big dog” when there is a small one as well), or assigned with respect to a referential domain that is tied to a local goal. For example, when completing a jigsaw puzzle, “try the red piece,” might be uttered—and easily understood—in a collaborative task, when there are several pieces that might fit and several other red pieces that are clearly the wrong size and shape (Brown-Schmidt and Tanenhaus, 2008). A central question, then, in both reference generation and comprehension is how interlocutors choose a specific referential expression and then modify its use as a discourse unfolds.

In a classic paper, Brennan and Clark (1996) introduced the notion of a *conceptual pact.* Building on the foundation established by Clark and Wilkes-Gibbs (1986) and Isaacs and Clark (1987) in developing the collaborative model of conversation, Brennan and Clark argued against what they termed “a historical” accounts of the generation of referring expressions. They proposed that when participants agree upon a name, that is when the name has been *grounded*, they are making a *temporary* agreement about how to conceptualize that object. The aspect of conceptual pacts that has received the most attention in recent years is the claim that conceptual pacts are partner-specific. For example, there is an ongoing debate about how and when the use of a particular name for an object will affect processing, specifically when an established name (a maintained precedent) or a different name (a broken precedent) is used by the same partner or by a different partner (see Kronmüller and Barr, 2015 for a recent review and meta-analysis).

The current research focuses on an important aspect of conceptual pacts that has been largely ignored in the literature. In comparing their proposal with pioneering work by Carroll (1980, also see Carroll, 1985), Brennan and Clark noted that a defining characteristic of their model is that conceptual pacts are temporary and adaptable. They argued that participants can modify their utterances abruptly, that is without negotiation in response to changing goals and informational needs. Thus when the interlocutors’ goals change or when the informational demands of the local context change, then the pressure to adhere to a conceptual pact might be relatively weak. If that is the case, then elucidating these factors will be crucial to developing robust theories of reference generation and understanding. Here we ask (1) whether interlocutors do indeed abruptly change their referential expressions–in particular negotiated names–when either the goals or informational demands change; and (2) whether frequent use of a name in a conceptual pact causes the name to become entrenched, that is resistant to being changed.

Given the focus in the literature on the benefits of maintaining precedents and the costs of breaking precedents, Brennan and Clark’s (1996) evidence for conceptual pacts becoming entrenched is arguably quite weak. They found that when interlocutors used a subordinate-level name because a picture was introduced in the context of another member of the same category (e.g., a collie in the context of two dogs), they often continued to use that name rather than the more basic-level name (e.g., “dog”). This can be viewed as a form of over-modification, which is quite common (see Pogue et al., 2016).

Conversely, the evidence for the flexibility of conceptual pacts is also relatively weak. Brennan and Clark (1996) found that after using a basic-level name, such as “shoe,” interlocutors would switch to a subordinate level category, e.g., “penny loafer” when confronted by a situation in which there was more than one type of shoe. In this situation each of the potential referents would be an equally good fit to the previous referential description of “shoe.” Moreover, changing the name from a basic-level category name to a subordinate-level name does not require a major shift in conceptualization.

If a grounded name reflects a *temporary* agreement to conceptualize an object in a particular way with respect to a *particular set of goals and informational needs*, then when the goals or informational needs change, participants will no longer seek to use previously grounded referring expressions. The current research was designed to test this claim using stronger manipulations than those used by Brennan and Clark (1996). In Experiment 1, we introduce a change in the goal structure of a collaborative task that could potentially trigger a bigger shift in conceptualization in order to see whether or speakers will make non-negotiated, abrupt changes in their use of a referring expression. In Experiment 2 we introduce a change in the referential context by introducing a new object that could be treated as contrastive or not, depending on whether participants choose to maintain a previous conceptual pact.

Both experiments use “targeted language games” (Brown-Schmidt and Tanenhaus, 2008; Tanenhaus and Brown-Schmidt, 2008) with task-oriented dialog. In most experimental work on naming, the referential domain depends primarily on a fixed set of potential referents, with little or no collaboration and minimal task constraints (e.g., Nadig and Sedivy, 2002; Sedivy, 2003; Brown-Schmidt and Tanenhaus, 2006; Engelhardt et al., 2006; Brown-Schmidt and Konopka, 2008). Thus there are no changes in either the goals or informational demands that might lead participants to abandon previously established conceptual pacts.

In task-oriented dialog, however, interlocutors work together to establish a given name in order to uniquely identify a referent with respect to goals, which might be hierarchically arranged. Therefore the language is unscripted and the referential domains are likely to be more fluid than in studies using other tasks.

An example of how task constraints and local goals can modulate referential domains comes from a study by Brown-Schmidt and Tanenhaus (2008). In their study, two participants who could not see one another collaborated to match place pieces on their respective Duplo^TM^ pegged boards such that the boards would match. Participants had identical boards that were divided into several sub-regions defined by cardboard borders. Additionally, participants had stickers covering the pegs on the board indicating the type of block (e.g., color and shape) that was to be placed in that particular location; where one participant had a sticker, the other participant’s board was blank. Brown-Schmidt and Tanenhaus analyzed utterances in which one of the participants referred to a block when there was a potential competitor referent in the same sub-region (e.g., a red, vertical block, when there was another red block, like a red horizontal block). Whereas one might expect speakers to use referential expressions that would take into account all of the potential referents, more than 50% of there referential descriptions were “under-informative,” For example, the speaker might say “Put it above the red block.” Surprisingly, these potentially ambiguous referring expressions were not confusing to the addressee. When the task constraints were factored in, these “under-informative” referring expressions did in fact uniquely identify a single block, (e.g., only one of the red blocks had enough space above it to put another object). The goal of placing blocks in particular configurations thus restricted the referential domain to only those that fit.

We explore two hypotheses about the context-dependence of conceptual pacts. The first hypothesis is that a conceptual pact is specific to a task goal. The strongest form of this hypothesis is that when the goal changes, interlocutors will neither seek to maintain, nor avoid breaking, lexical precedents. The second hypothesis is that participants will no longer be bound by prior conceptual pacts whenever there is a change in the potential informational demands. We assess this by introducing new objects that could be named in different ways to see if participants seek to generate a referential description that would allow them to maintain a lexical precedent.

## Experiment 1: Conceptual Pacts Amid Shifting Goals

In Experiment 1 we created a situation in which two interlocutors who could not see each other’s work areas collaborated to build a three-dimensional puzzle of an animal. The partners’ task was divided into two phases. In the first phase, participants retrieved and named relevant puzzle pieces (abstract shapes) from a larger set using instructional cards as a guide. Each card showed a picture of an object that would be needed later. Participants had no knowledge of the end goal of the puzzle. In the second phase, participants used those pieces to build the animal puzzle. We focused on whether the shift in goal from picking out abstract objects from a set to the assembly of an animal would predict a change in references to objects with previously grounded referring expressions. We asked whether an abstract object referred to by a grounded name (e.g., the wrench) would be referred to by a different, object-based name (e.g., the leg) when the goal is shifted, and if so, whether the change in name would need to be negotiated or even commented on by the interlocutors (e.g., “the wrench is a leg”). We also hoped to find cases where that piece did not fit (e.g., the piece referred to as “the leg” wouldn’t fit) in order to see whether participants would then reuse the negotiated name.

### Materials and Methods

We created a turn-taking language task designed to elicit repeated reference to a specific set of objects. The design is a subset of a larger paradigm used in studies assessing linguistic convergence and miscommunication (Roche et al., 2013; Paxton et al., 2014). The participants’ overall task was to build three-dimensional puzzles using pictorial instruction cards. The task constrained both the objects requiring linguistic reference and the order in which they were to be manipulated, but participants were otherwise able to speak freely. In addition, a barrier was introduced so that there was no shared visual space, thereby necessitating that using spoken language was the primary means of communication (e.g., shifts in gaze or point could not be used as cues). Turns were implemented rigidly, as instructions were staggered across the two instruction stacks, one for each participant and both of which made up the full set of instructions. These were ordered to ensure successful alternation of turns.

**FIGURE 1:**
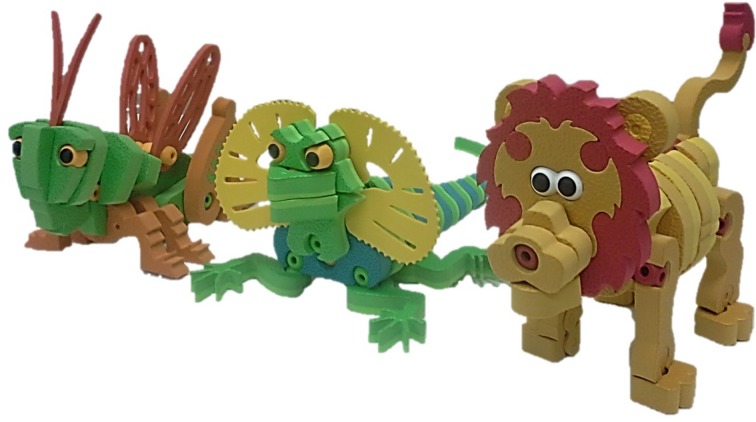
**Bloco^TM^ objects: grasshopper, lizard, lion**.

**FIGURE 2:**
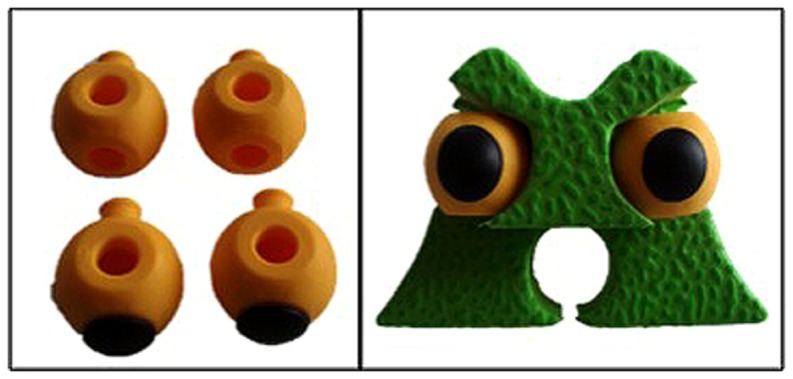
**Sample item phase instruction card (left) and build phase instruction card (right) for lizard**.

Stimuli included three Bloco^TM^ animal puzzles, consisting of a grasshopper, lion and lizard (see **Figure 1** for final product) and order of objects was counterbalanced across participants. The number of instructions varied between the objects (total range: 17–32 instruction cards; see **Table 1** for a breakdown; see **Figure 2** for sample instruction cards).

**Table 1:** Number of instruction cards for each animal, total and by phase.

Object	Total instructions	Item phase	Build phase
**Lizard**	17	9	8
**Grasshopper**	23	10	13
**Lion**	32	15	17

#### Task: Phase Structure

To create a situation in which repeated reference to the same objects would occur across two different contexts by virtue of a shift in task, separate phases were introduced: the *item phase* and the *build phase*. In the item phase, participants picked out the individual pieces needed to build the object. Both participants were given identical sets of pieces in a bin. During a turn, each participant would pick out the piece on her card from her bin and then describe the object so that her partner could do the same. Instructions alternated until the proper subset of items had been selected. Note that the identity of the object to be built was not given until the item phase was complete.

Once the item phase was complete and the participants had matching pieces on their workspaces, the build phase began. During the build phase, participants also alternated instructions, with each instruction card describing how to build a combination of those pieces obtained in the item phase; these were ordered and staggered to establish turn taking. Crucially, these pieces were to be combined and then added onto a pre-constructed object—an unfinished animal body. The unfinished animal body was presented at the beginning of the build phase in order to emphasize the shift in task. In the item phase, we expected participants were likely to uniquely identify pieces relative to the set of alternatives in the bin. In the build phase, we expected that participants would refer to pieces relative to their relationship to the build-object, especially once its structure began to emerge.

For both phases, participants were video-recorded and the audio was extracted to fully transcribe the participants’ dialog. Transcriptions were then coded with the following annotations.

#### Annotations

Discourse features:

(1) Speaker identity: the identity of the speaker at any given turn.
(2) Local shifts in task: tracking of trial; each instruction card representing a trial, constrained by task and specific object.
(3) Global shifts in task: tracking of phase; either item or build.

Features of referential expression:

(1) Referent: the object being referred to by the referential expression.
(2) Groundedness: presence of grounding expression following referential expression.
    
*Fine-grained*: classed individually as “mhm,” “yup,” “yeah,” “yes,” “okay,” “alright.”
    
*Course-grained*: classed as “mhm,” “yup,” “yeah,” “yes,” “okay,” “alright.”
(3) Definiteness of description: presence of a definite article preceding description.
(4) Distance from previous mention: number of turns between repeated references to same object, semi-constrained by task.
(5) Noun phrase head: head noun of referential expression.
(6) “Like” complement: referential expression following a “like” construction, most commonly a “looks like” construction.

#### Outcome Variables

The variable of interest was a change of a referential form, as realized in the repeated reference of any given puzzle piece. This was operationalized as a change in the head noun of a referring expression.

**FIGURE 3:**
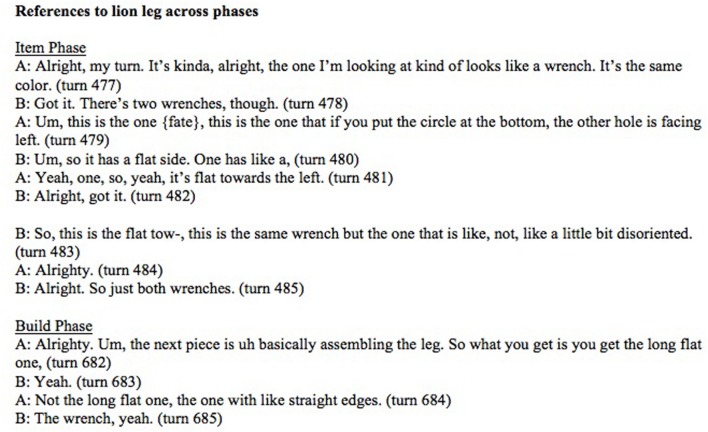
**Transcript of participants referring to a puzzle piece that will become the lion leg in the build phase (Pair 4)**.

Secondarily, the specific type of change was explored, with any given change categorized as either a *negotiated change* or an *abrupt change*. We operationalized negotiated changes as those referential expressions whose changed form was introduced obliquely in prior discourse but not as the head noun. Because these often were the complements of constructions such as “looks like a wrench,” this was the only linguistic construction used to code negotiated changes. This was a simplification, as changes in form might be negotiated in discourse in a different way, however, for present purposes, we restricted negotiated instances to the “like-complement” cases. Abrupt changes were taken to be any change in head noun whose new form was not given in previous discourse. See **Figure 3** for an excerpted transcript of participants who call a piece “the wrench” repeatedly in the item phase but shift to calling it “the leg” without overt negotiation.

#### Participants

Nine pairs of undergraduates participated from the University of Rochester (*N* = 18). All participants were native speakers of American English with normal to corrected vision. None reported speech or hearing impairments. This study was carried out in accordance with the Research Subjects Review Board (RSRB) at the University of Rochester. All participants gave written informed consent in accordance with the Declaration of Helsinki and were debriefed and offered a copy of the consent form upon completion of the experiment.

### Results

#### Descriptive Data

The following description and analysis includes data from the nine pairs of participants. The dataset consisted of a total of 1,776 turns containing referential expressions. These referential expressions were hand-annotated and then coded for *groundedness*. A referential expression was coded as grounded if it was followed by any of the following agreement markers: *mhm*, *yup*, *yeah*, *yes*, *okay*, or *alright*. All other referential expressions were coded as not grounded. A non-grounded expression, if taken to be a proposal for a name that has not been accepted, is likely to undergo changes until a name is established. This was not a change in form that we were interested in, since they would not be broken pacts. Therefore, we restricted our dataset to grounded expressions. These made up the subset of data that was used for the following analyses. **Table 2** presents a breakdown of the number of grounded and changed referential expressions. Note that when looking at head noun changes generally, they were quite evenly distributed across groundedness: 50.58% of head noun changes were grounded. Thus changes were not primarily the result of a pact not having been formed in the first place.

**Table 2:** Total number of referring expressions.

Total referential expressions	Grounded	Changed
871	429	173

*Note: A subset of the total were grounded, and a subset of the grounded expressions underwent a change in form (nested values).*

#### Analyses

**FIGURE 4:**
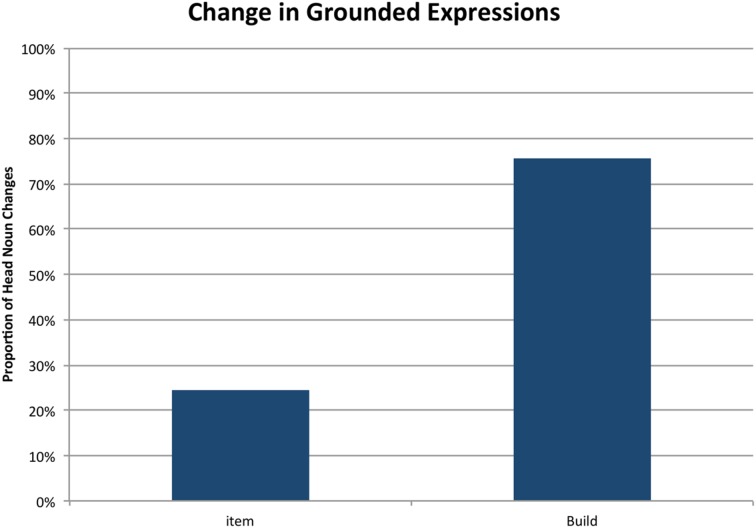
**Proportion of changes in grounded referential expressions across phases**.

To assess whether a context shift predicted a change of form, we used a generalized linear mixed model including random intercepts only, with presence of head noun change as the outcome variable, phase as predictor and pair as a random effect. The model showed that build phase significantly predicts a change in form (β = 0.86, *SE* = 0.13, *p* < 0.001). **Figure 4** presents the proportion of changes in the two phases.

**FIGURE 5:**
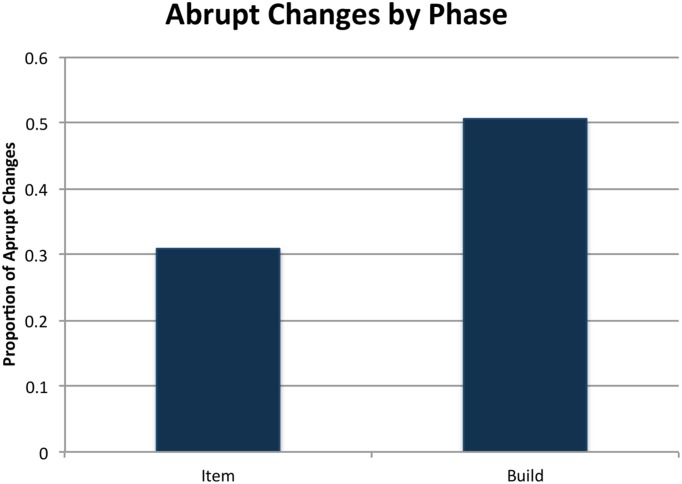
**Proportion of abrupt changes in grounded referring expressions within each phase**. Negotiated changes are the converse.

A second generalized linear model was used to assess how changes were realized across a task-goal shift. We included random intercepts and the model contained phase as the outcome variable, change type (either negotiated or abrupt) as the predictor and pair was the random effect. The decision to reverse the directionality of the model from the first was motivated by the fact that the categories of change type were *ad hoc* categories meant to provide a finer-grained description of a change in form. Thus, we wanted to assess whether occurrences of a particular type of change could predict whether the pair was in the item or build phase. Indeed, the model shows that an occurrence of an abrupt change increases the likelihood that a pair is in the build phase (β = 0.975, *SE* = 0.23, *p* < 0.001). See **Figure 5** for the proportions of abrupt changes for each phase. Although there were not sufficient trials for a statistical analysis, we observed a striking phenomenon that further highlights the context-dependence of names. On 16 trials, one of which is illustrated in **Figure 3**, participants made an abrupt change from a negotiated name (e.g., *wrench*) to an object-oriented name (*leg*s) and then found the piece would not fit. On each of these trials, participants then reverted to the name used during the item-phase (e.g., wrench). This illustrates that participants can shift seamlessly between different names, which reflect different conceptualizations of the objects tied to different task goals.

### Discussion

In Experiment 1, changes in the context were instantiated by a shift in task goals, i.e., the shift from the item phase to the build phase. This allowed us to determine whether a conceptual pact that was established when the participants had a particular goal would need to be renegotiated when the goal changed. The answer was clearly “No,” highlighting that conceptual pacts are strongly dependent on task goal. We note that an overall task goal, such as building an animal, is likely to have hierarchically organized subgoals, e.g., building a part of the animal (e.g., the face). We are assuming the conversation takes place against the backdrop of these goals as well as more local subgoals. This raises the important issue of how local task goals interface with a conversation as it unfolds. One possibility is that local goals might play a role in determining the Question Under Discussion (Roberts, 1996), but this is beyond the scope of the current paper.

In Experiment 2, we examine the extent to which a name agreed upon in a conceptual pact is entrenched when new objects are introduced that may potentially change the informational demands. First, we explore whether negotiation of a name is predicted from the properties of an object, i.e., whether it is a commonly identifiable object given the alternatives. This manipulation was important for two reasons. First, it allowed us to establish that the typical pattern for negotiated referring expressions, namely a reduction in length across repeated references would be replicated in our task. Secondly, increased negotiation for less identifiable objects would provide evidence that that object is likely to be more compatible with multiple descriptions compared to the more identifiable objects. As will become clear this property of our design creates situations in which the speaker could generate a referential expression that would allow them to maintain a lexical precedent.

## Experiment 2: Conceptual Pacts Amid Emergent Naming Competitors

We created a targeted language game (Brown-Schmidt and Tanenhaus, 2008; Tanenhaus and Brown-Schmidt, 2008) modeled on puzzles in which a player moves tiles in a constrained space to match a target pattern. Participants were tasked to move the tiles collaboratively to achieve the target configuration. Crucially, three of the tiles were occluded and were only revealed after they had been moved to a particular location. In the critical case, an object that could be identified with a name or a description (e.g., a tangram animal) is occluded and later revealed, allowing for the establishment of a referring expression for the already-visible and more prototypical picture (e.g., a clip art animal). This is contrasted with the reverse order, in which a prototypical picture is visible and a tangram is revealed. Adherence to a conceptual pact was assessed by measuring rate of modification. Unlike in Experiment 1, this set-up calls for the goal and task structure to remain the same and instead assesses the effects of potential changes in information demands. In particular we ask: (1) whether a referential expression for an old object will be modified after encountering a new object and (2) whether the frequency of use, or extent to which a name has been negotiated, affects whether participants continue to use the old name.

### Methods and Materials

**FIGURE 6:**
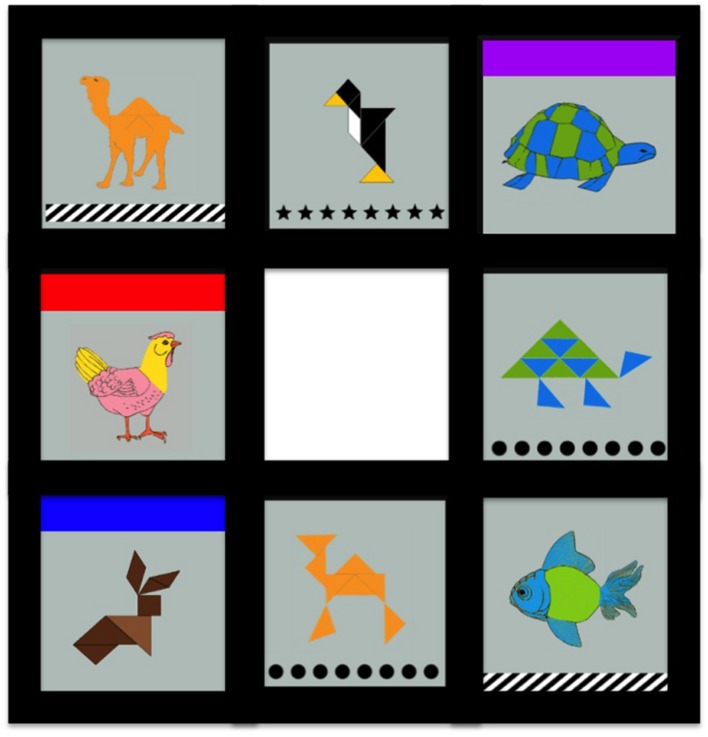
**Game board with four tangram images and four clip art images**.

The game board consisted of a fixed three by three grid. Each cell contained a game tile except for the middle cell, which was empty. **Figure 6** presents a sample game board. Tiles can only be moved into the empty square. Movements into the empty space were possible only for adjacent tiles (e.g., it was invalid to move from top left corner to bottom right corner). The target configuration required the matching of colors and patterns as described below.

#### Animal Cards

**FIGURE 7:**
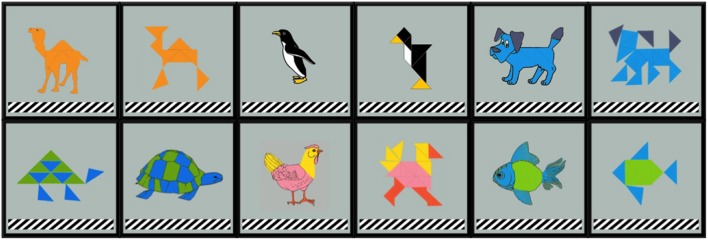
**Side by side comparison of tangram and clip art animals**. A subset of six is shown.

Each game tile in the occupied cells contained a depiction of an individual animal in its center. Half of the cards (i.e., four of the eight) had depictions that were tangrams, or geometric figures, that resemble an animal. The other four cards had more-identifiable clip art animal depictions. To create an image bank of animal graphics of both types, tangram images were used as a base and modified in an image editor by “photo-shopping” in clip-art details to create the more identifiable versions, maintaining color and size consistency in the process. The full image bank consists of 14 animals each with two versions: tangram and clip-art/modified. These are hereafter referred to as *potential naming competitors*. **Figure 6** shows this mix of item types in a sample game board and **Figure 7** illustrates side-by-side comparison of image type. This feature of the game emerged from a starting observation: it is unclear to what degree the entrenchment of a name depends on properties of an object. That is, if partners collaboratively form a conceptual pact to refer to an object by a specific name, the process by which that name is agreed upon might be different depending on the identifiability of the object. By adding tangram versions of items in this game, we created a condition where those objects were not only likely to require more negotiation to establish a name, but could be referred to in different ways due to not being good exemplars of a particular animal.

#### Two-Player Adaptation

This game was adapted for two people in the following ways. First, players only have visual access to their own individual game board. They sit face to face with their partners, but each has a computer screen the other cannot see. Secondly, players must play together but make move in alternating order, with one player making a move and instructing her partner to do the same and vice versa. Crucially, each player’s game board only has partial information of what is needed to achieve the target configuration: both players’ game tiles have the same animal depiction, but there is also accompanying card information that is either displayed in the form of a *color bar* or a *pattern bar*, above or below the central animal. There are three possibilities: Color bars could be red, blue or purple; pattern bars could be stripes, dots, or stars. Furthermore, these bars appear in complementary relation. For example, if Player A’s chicken has a red bar on top, Player B’s chicken might have stars. Any other animal with a red bar for that game would have a counterpart with stars. Players do not know which color corresponds to which pattern, so they are likely to refer to a game tile by the shared information, i.e., the animal name. Furthermore, displays for both players were mirror images to allow for the same moves while discouraging location-based referring expressions.

In sum, then, the participants’ task was to configure the three corresponding red/star animals in one row, the three blue/dots in another and the two purple/stripes in the last row, with the exact configuration left up to the players.

#### Occluded Tiles

As described above, eight tiles had animal images, but only a subset was visible from the start of the game. In particular, three of the eight tiles initially were hidden by an opaque colored square that covered the animal and color/pattern information. These occluded tiles were revealed when they were moved to the (empty) middle square. Once the occluded tile was moved to the center, the game tile was revealed and remained visible throughout the rest of the game. Of most importance, for the critical game, two of the occluded game tiles covered animal images that were potential naming competitors to animals on the game board: one was a hidden tangram version, the other a hidden clip-art version. The third occluded tile covered a singleton animal to discourage participants from becoming aware that there might be a tangram/clip-art contingency. This allowed for participants to establish names for the visible objects before encountering an alternative that might plausibly be treated as a contrast. More specifically, it created a situation in which a tangram object that after negotiation had been called “the camel” might be moved, and thus referred to, several times before a more prototypical camel is revealed. This set up allowed us to address three questions within the context of an unscripted interaction among naïve participants.

The first question is to what extent a name remains entrenched after a new referent is introduced that could be conceptualized as a contrast to a previously named referent. If the introduction of a naming competitor results in a modification to an established name, this suggests a new conceptualization of the original object, and more importantly, the flexibility of a conceptual pact to accommodate new contextual information. The second question is whether the type of competitor would predict the likelihood of modification. That is, if the revealed object were a tangram, would its name likely be negotiated to a common name without appeal to a contrast set? Similarly, would a hidden clip-art image elicit more modification given that its name is more commonly shared? The third question is whether the name for an object becomes more entrenched with repeated use, i.e., whether it becomes more resistant to modification.

#### Conditions

Each pair played a total of two games. The first game had all singleton items with no naming competitors occluded or visible. The second game had the crucial occluded tiles covering the tangram and clip art counterparts to visual tiles, as well as a singleton.

To manipulate the relative strength of a conceptual pact we presented the three conditions to also assess carryover across each game, as follows. Game 1 contained all singleton items. Game 2, on the other hand, had counterpart items as well as potential carry over from Game 1. The reason for only including singleton items in Game 1 was to prevent participants from beginning Game 2 with the assumption that occluded tiles might contain potential contrast items.

In the *No Carryover* condition: Game 1 had no items that carried over to Game 2.

In the *Carryover Strong* condition: Game 1 had two singleton items that carried over to Game 2. In Game 2, these singletons were visible and their naming competitors, never before seen, are occluded. This was to encourage a strong conceptual pact for the name given in Game 1.

In the *Carryover Mediated* condition: Game 1 was identical to Game 1 of *Carryover Strong* condition. The same two singleton items carried over to Game 2, however, the items previously seen were the naming competitors that were occluded. They were only revealed later in the game. This is designed to test for reuse of common names for different referents at the start of Game 2.

Annotations for all token referential expressions were isolated and coded for length of expression in number of words, modification (presence/absence), negotiation (presence/absence), naming competitor status (had competitor/did not), ordering (hidden/visible). One pair’s Game 1 was not included in the analysis due to a technical error: recording of the Game 2 dialog erased the recording of the Game 1 dialog.

#### Participants

Fifteen pairs of participants were recruited from the University of Rochester (*n* = 30). All participants were native speakers of American or British English with normal to corrected vision. None reported speech or hearing impairments. One participant reported color-blindness. This study was carried out in accordance with the RSRB at the University of Rochester. All participants gave written informed consent in accordance with the Declaration of Helsinki and were debriefed and offered a copy of the consent form upon completion of the experiment. Speech was recorded and transcribed as a full corpus similar to Experiment 1.

### Results

For purposes of the analyses reported here, we collapsed across the three “carryover” conditions. In follow-up work with more pairs, we will examine the effects of these conditions. There were 1671 total referential expressions referring to the animals on the game cards. References to the occluded squares and location-based references to the game cards were omitted from the count. Both occluded squares and location-based references were often referred to with modification (e.g., “the green occluded,” “the bottom middle,” respectively), which would artificially increase the modification rates of interest. The latter were omitted also because these references had different referents for each partner. Because game boards were displayed as mirror images, participants who used location-based references were often likely to miscommunicate and would only realize later in the game that their partner was not moving the same card she moved. These occurred rarely, except for one pair who did not understand the goal of the game and used these references extensively in Game 1.

#### Negotiation

**FIGURE 8:**
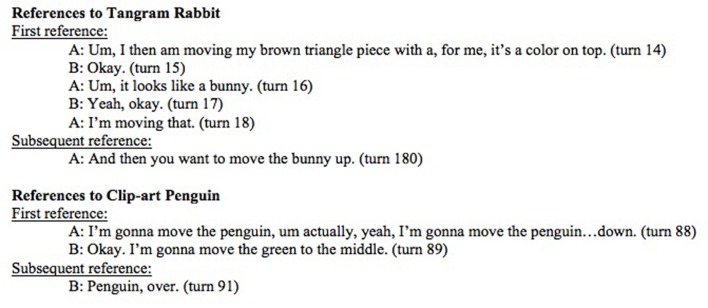
**Transcript of participants playing tangram/clip-art game (Pair 6).** Negotiated vs. non-negotiated name use.

Tangram objects were more likely to have names that required negotiation than others. Of the 113 referential expressions that were negotiated, 91.2% were tangrams. See **Figure 8** for a sample of a transcript illustrating one instance of negotiation for a tangram animal but no negotiation for a clip-art animal. In order to measure the strength of association between item type (i.e., whether a card was a tangram or a clip-art graphic) and negotiation status (i.e., a binary yes or no), we used a logistic regression model with negotiation as the outcome variable and item type as a predictor. Results indicate that tangrams have 11.31: 1 odds of negotiation relative to clip-art items (Wald-statistic = 7.24, *p* < 0.0001), with the overall model significant (*p* < 0.0001).

#### Length of Referring Expression

**FIGURE 9:**
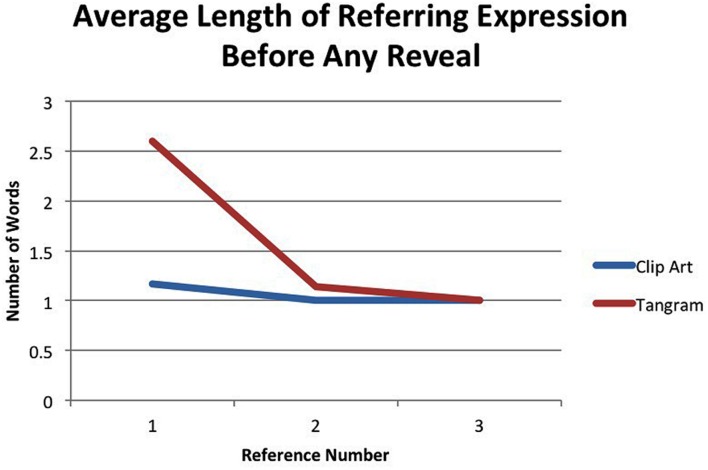
**Average number of words for the first three references prior to the reveal of a new referent**.

To assess changes in referring expression, we first measured length of referring expression. We asked whether the utterances in our tasks exhibit the standard behavior of repeated reference in past studies (e.g., Carroll, 1980; Clark and Wilkes-Gibbs, 1986; Brennan and Clark, 1996). As interlocutors converge on a name, subsequent references should become shorter. **Figure 9** shows the average length of the first three references to an object with a competitor *prior* to the reveal of its competitor. A significant difference of length is shown between clip art and tangram items on the first reference but by the third reference, the average length of referring expression for both item types has converged to one word (e.g., a bare noun). We fit a generalized linear model with length as the outcome and item type and reference number as the predictor. There was a main effect of both item type and reference number: tangrams were found more likely to have longer lengths overall (β = 0.723, *SE* = 0.2843, *p* < 0.05), and crucially, as references increased, length decreased (β = -0.4539, *SE* = 0.1846, *p* < 0.05).

**FIGURE 10:**
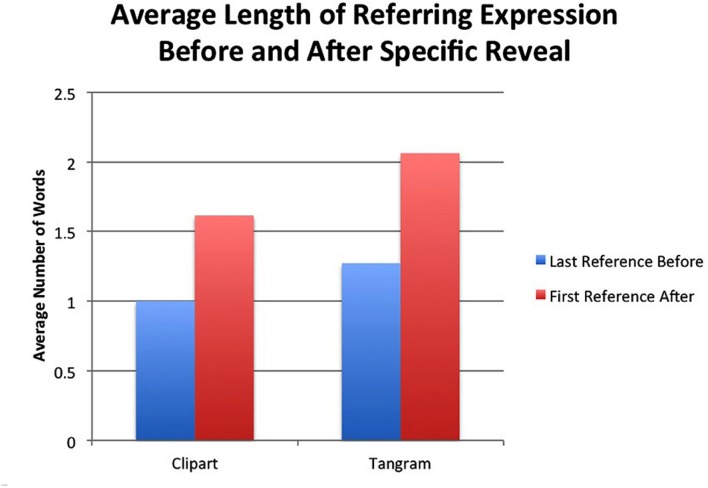
**Average number of words in referring expression before and after a specific competitor item was revealed**.

Next, we assessed length of referring expression before and after the reveal. After the reveal of a competitor item, one might see a similar reduction of the length of the referring expression, indicating further convergence on a name. However, if a participant conceptualizes the revealed object as a contrast item, the length of the referring expression might plateau or even increase. We did a contingency analysis for items with a potential contrast and fit a generalized linear model with length as the outcome and item type and before-and-after reveal status as predictors. There was no main effect of item type (*p* = 0.13) but a main effect for reveal status. That is, for both clip art and tangram objects there was an increase in length after the reveal of a potential contrast (β = 0.7065, *SE* = 0.2462, *p* < 0.01). **Figure 10** shows the average length of referring expression for the single reference before and after the reveal of its competitor.

**FIGURE 11:**
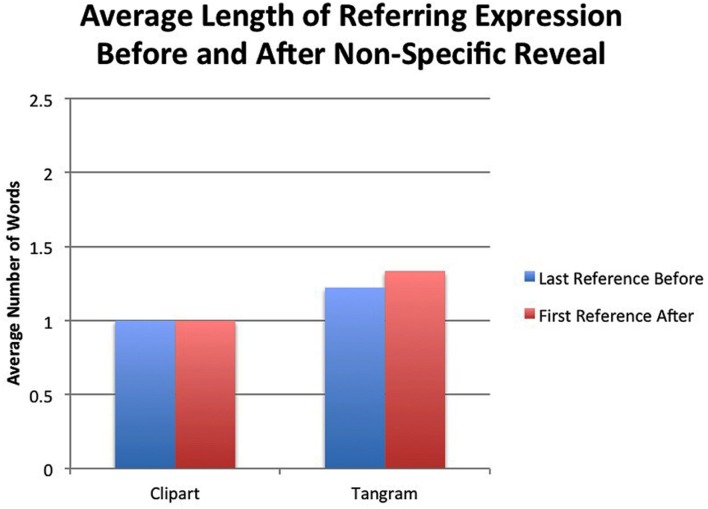
**Average number of words in referring expression before and after a non-specific item was revealed**.

Length around the reveal of a non-specific item (i.e., an item that is not a competitor) was also assessed. **Figure 11** shows the average length of referring expressions for items that would eventually have a competitor, before and after an item was revealed that did not have a potential contrast, which we will term a non-specific item. These non-specific reveals occurred prior to the specific competitor reveal. For clip art items, there is no increase in length, and for tangram items, there is a numerical but non-significant increase. This suggests that the increase in length is particular to a potential contrast and not a new object in the display. We next assess whether this increase in length could be explained by modification.

#### Modification

##### Modification: base rate

Base rate modification was low overall, with 22.4% of the 1671 references having modified referential expressions. Of the 374 modified referential expressions, 62.8% were tangrams, regardless of whether a naming competitor was present in the visual display. Looking at Game 1 only, the modification rate across *all* item types was 28.3%. For Game 2, the modification rate for those items without a naming competitor was 17.4%, but this rose to 54.3% when the items had a potential naming competitor.

##### Modification: before and after reveal

**FIGURE 12:**
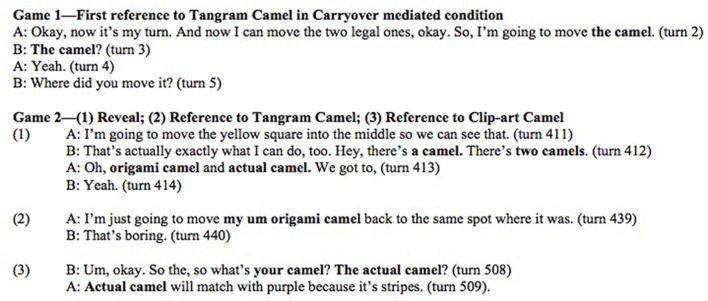
**Transcript of participants modifying referential expressions for both camels in Game 2, despite using common name in Game 1 (Pair 7)**.

As stated above, there were three types of items that were occluded: a tangram with an occluded clip-art competitor, a clip-art with an occluded tangram competitor, and an occluded singleton. The proportion of modification for all competitor items *before* its competitor was introduced was 19.6%, whereas the proportion of modification for competitor items *after* its competitor was introduced was 53.2%. See **Figure 12** for an excerpt of a transcript in which an item previously unmodified is modified after the reveal of the naming competitor. Breaking down the data by item type, tangrams were modified 31.3% before the reveal and 69.4% after the reveal. Clip art images were modified 6.7% before the reveal and 35.8% after the reveal.

The crucial question was whether aspects of the revealed competitor affected entrenchment of a conceptual pact. Looking only at those occluded items with competitors, we used a generalized linear mixed regression model to measure the extent to which item type, number of “before-the-reveal” references, and order of reveal predicts modification. We used modification as the outcome variable and included four predictors: reveal state (i.e., before or after the reveal), item type (tangram or clip art), order of competitor reveal (whether tangram or clip-art was the occluded item), and number of “before” references. A random effect of pair was also included in the model. We introduced both random intercepts and slopes and removed one at a time until the model converged. The model shows that “before-the-reveal” references were less likely to be modified than “after-the reveal” references (β = -2.06, *SE* = 0.43, *p* < 0.001). In addition, tangrams were more likely to be modified (β = 1.57, *SE* = 0.24, *p* < 0.001) than clip art images. However, neither the order of the reveal nor the number of “before-the-reveal” references was significant (β = -0.45, *SE* = 0.31, *p* = 0.15 and β = -0.07, *SE* = 0.09, *p* = 0.48, respectively). Crucially, then, the pressure to maintain a lexical precedent for the clip art image, which was a better fit to the established name, was not sufficient to lead interlocutors to choose a non-contrastive description for the potential tangram competitor.

### Discussion

The results of Experiment 2 show that conceptual pacts are easily broken when information demands change, even when names have been negotiated and used multiple times. We found clear evidence that names converge and reduce in form with repeated references, replicating previous results. Convergence was noted for both tangram and clip art images suggesting that despite differences in goodness of fit, the naming process was similar. Initial references to tangrams were longer than initial references to clip art images. However, by the third reference, both had converged to the length of a bare noun. The surprising result is that length increases equally for both the tangram objects and the clip art objects after the reveal of a specific competitor, suggesting a breaking of the conceptual pact, even when for a tangram-reveal the speaker might have chosen a name that did not change the name of previously mentioned clip art.

The increase in length was corroborated by an increase in modification. However, there was a higher modification rate overall for tangram objects than clip art. This asymmetry suggests that the images have different goodness of fit with the presumed negotiated name. Accordingly, these images are not obvious contrasts with each other, but could still be conceptualized as such. Although the overall modification rate is higher for tangram objects, both objects are modified after the reveal, and crucially the effect of introducing a potential competitor on modification is not contingent on a particular order of reveal. Thus, modification, here, was tied more closely to competitor presence than the type of competitor. Furthermore, the number of references made prior to the reveal did not predict modification, suggesting that frequency of use of a name is not associated with that name becoming more entrenched. This supports a strongly context-dependent view of conceptual pacts, one in which pacts are flexible enough to accommodate as a new conceptualization of an object, which may occur at any point in the interaction.

## General Discussion

A conceptual pact has historically been framed as a temporary agreement between interlocutors to not only refer to objects by a particular name but also to adopt a particular conceptualization of the referent (Brennan and Clark, 1996). Although Brennan and Clark (1996) emphasized the temporary, context-specific nature of conceptual pacts, that claim had not been well-established in the literature. In fact the focus on the costs of breaking a lexical precedent suggested that there are strong pressures for interlocutors to maintain a conceptual pacts. To evaluate the context-dependence of conceptual pacts we created conversational interactions in which either the task goals or the potential information demands of the referential domain changed. We asked whether interlocutors would maintain pacts when the local context changes. We found little or no evidence that interlocutors felt bound by names, even negotiated names, when there was a change in these contextual features.

Experiment 1 focused on the extent to which conceptual pacts are bound to specific task goals. We used a puzzle paradigm to elicit unscripted references to objects across different goals with a single partner. Furthermore, we designed the experiment to elicit multiple references to objects in order to assess the possibility of naming changes over time. The variable of interest was not only whether changes occurred but, if so, how they occurred. That is, would a new name for an object require renegotiation? Or would the new context created by the change in the goal structure, from identifying abstract pieces to using those pieces to build an animal, result in abrupt changes in referential descriptions? Indeed, we found that conceptual pacts were easily abandoned when switching from a phase of a game that required identifying single pieces to a phase that involved assembling those pieces into an identifiable animal. Participants were likely to change a name that was already grounded. Even further, they were likely to change that name to an animal-based name abruptly without negotiation. Moreover, interlocutors would return to the previous negotiated name when the action based on the animal-based reference was unsuccessful, i.e., when the parts assumed to be a leg would not fit.

Experiment 2 focused on the degree to which names are entrenched in the face of changing referential alternatives. In this experiment, change was explored more narrowly by measure of length of referential expression and, specifically, modification to an established name. Modification has been shown to be a way of lexically marking contrast in a referential domain (e.g., Nadig and Sedivy, 2002; Sedivy, 2003; Engelhardt et al., 2006; Brown-Schmidt and Tanenhaus, 2006; Brown-Schmidt and Konopka, 2008). And importantly, it connects back to the original work on conceptual pacts (Brennan and Clark, 1996), which used over-modification as a way of determining adherence to a name established in the presence of a contrast set. One example is calling particular footwear in an array “a dress shoe” in the presence of other types of shoes and then maintaining this over-specific form when encountering the object in an array where there are no other shoes. This is taken as evidence that conceptual pacts are maintained (i.e., names are entrenched) despite the change in visual context. However, over-modification is often taken to be more acceptable in conversation as under-modification for various reasons (see e.g., Isaacs and Clark, 1987; Pogue et al., 2016), which reduces the strong claim that name entrenchment and conceptual pacts are creating pressures to use overly specific forms.

Experiment 2 used a targeted language game to create a situation where participants used a name, negotiated or otherwise, and then were presented with an item that may or may not be viewed as a member of a contrast set. This allowed us to examine the degree to which conceptual pacts become strengthened by increased repetition, thereby becoming more resistant to modification. We chose images (i.e., tangrams) that have precedent in language studies of reference and conceptual pacts. However, our modifications to the images allowed us to present a “naming competitor” that is not simply semantically similar but might better fit a common name already grounded. This asymmetry in fit allowed for either option to occur in conceptualization: participants could view them as pairs of a contrast set or choose not to given that one is a better exemplar. Visual similarity helped us avoid objects that might be contrasted by use of a prenominal adjective along a dimension such as size or color. Instead, we saw modifications like “the real camel” and “the origami camel.” By occluding some objects including potential competitors, we were able to create conditions where the number of uses of a name, both those that were negotiated and those that were accepted without negotiation, varied as a natural consequence of how the dialog unfolded. As expected, introducing a possible contrast increased modification rates for referential descriptions of objects that had been referred to previously. Somewhat surprisingly, though, was that there was no effect of number of mentions on modification rates. This suggests that even if repeated mention might strengthen a conceptual pact, the effect is not strong enough to affect how a new object will be conceptualized, which would be the case if the conceptual pact were resistant to modification.

Taken together the results suggest that interlocutors choose names that are highly dependent upon local task goals and informational demands, and these names can change rapidly as different aspects of the context change. One the one hand, this is not surprising. Use of referring expressions is highly fluid, as indexed by the many to many mappings that can be observed in repeated references to objects in a discourse. On the other hand, the fact that pacts are so fluid is surprising. We find strikingly little support for any effects of repetition of a name. Once the context changes, the referring expression is determined with respect to that context.

These results suggest that it will be important to embed investigations of reference generation and understanding in richer dialogs where it is possible to investigate the effects of complex goal structures in conversation. Also, incorporating dynamic referential domains in a given interaction that allows for a reconceptualization of alternative objects will help us further understand how goals and naming alternatives influence one another. Targeted language games provide a promising methodological approach for pursuing these investigations.

These results also have implications for computational models of reference generation. For example, existing models have focused on the grounding process (e.g., Heeman and Hirst, 1995), but have not taken into account the temporary nature of the conceptual pacts that are created during grounding. Thus small changes in a local goal might result in abrupt changes to previously grounded expressions. Moreover, evidence for miscommunication might result in returning to referential expressions that were tied to previous goals. There is also a tradition of modeling reference generation and understanding, building on classic work by Dale and Reiter (1995) as an incremental process that takes into account the objects in the referential domain, including the salience of their properties. But what objects are in the referential domain and what properties are salient will be highly fluid and strongly determined by shifting goals.

It is useful here to consider an analogy to work in vision in natural tasks. There is a tradition of modeling shifts in attention to regions of a scene as indexed by using properties such as visual salience derived by integrating multiple feature values at each position within a scene (i.e., saliency maps; see Koch and Ullman, 1985). Moreover, these models correlate with fixation probabilities during viewing of a scene when the observer is not given a particular task. As reviewed by Hayhoe and Ballard (2005, also see Salverda et al., 2011), however, feature-based salience turns out to be a poor predictor of gaze patterns when a participant is engaged in a well-defined task and needs to derive certain information from the visual input to successfully complete the task.

## Author Contributions

AI and MT together designed the study. AI performed the research and the data analyses. AI and MT together drafted and edited the manuscript.

## Conflict of Interest Statement

The authors declare that the research was conducted in the absence of any commercial or financial relationships that could be construed as a potential conflict of interest.
